# A Primer of Materia Medica for Practitioners of Homœopathy

**Published:** 1892-05

**Authors:** 


### BOOK NOTICES.

A PRIMER OF MATERIA MEDICA FOR PRACTITIONERS OF HOMŒOPATHY. By Dr. Timothy Field Allen. Philadelphia: Bœricke & Tafel. 1892.

Here is an octavo volume of four hundred pages which is designed, as stated in the preface, "to present the characteristic features of the most important drugs used by homœopathic physicians. It may serve to refresh the mind of a physician when away from his complete symptomatology; it will help him discriminate when studying an unfamiliar pathogenesis."

On looking over the work we find present the old familiar key-notes or characteristics of the various remedies. We think it would have been advisable to have had these characteristics put in larger type than the general text to call the attention of the reader to their importance. This criticism is made because we are surprised to find that the rising generation of medical men are decidedly ignorant of these characteristics, many of the college faculties ignoring them as of no account. In this book a few of them are missing. Thus, under Sulphur, we do not find flushes of heat, nor weak, gone feeling in the stomach. Now these two symptoms have been verified so often, the recognition of them in severe cases of illness and consequent prescription of Sulphur, has been attended by such happy results that we do not see how they could have been ignored. On the other hand, we find under Aconite the warning given that this drug should be prescribed in febrile conditions only, when there is the characteristic anguish, anxiety, restlessness at night and thirst. This is admirable. Aconite is one of the most abused remedies in the materia medica. Most of the family guides to homœopathic practice are written by men who are but ill acquainted with Homœopathy, and so we find that Aconite is always advised for everything, and especially for the slightest rise in temperature regardless of any other symptom. The public have gotten this idea implanted, and consequently they are forever dosing themselves with Aconite. Yet it is not the most frequently indicated remedy. The *mental anxiety, fear of death, prediction of death, red, hot face, restlessness and thirst at night in bed* must be present to make of it the simillimum. Therefore Dr. Allen has shown considerable accuracy in his caution about Aconite.

Another commendation can be given this book for the "word of caution" in the preface: "Do not use this book nor the 'pocket-book' [Bœnninghausen's] instead of a more complete symtomatology. These works are intended simply to be suggestive; especially is this caution needed as regards the use of the 'pocket-book;' *it is not to be used for isolated symptoms*, only to aid when a full picture of the patient is taken." * * * "This primer is designed to give the 'gist' of each drug rather than its symtomatology."

